# The Impact of Restrictive Abortion Laws on Medical Residency Applications and Healthcare Access: Implications for Maternal Health Equity

**DOI:** 10.1155/jp/6613889

**Published:** 2026-06-30

**Authors:** Brooke Gully, Ellie Whitney, Paige Johnson, Shanna Combs

**Affiliations:** ^1^ Department of Medical Education, Anne Burnett Marion School of Medicine at TCU, Fort Worth, Texas, USA; ^2^ Department of Obstetrics and Gynecology, Anne Burnett Marion School of Medicine at TCU, Fort Worth, Texas, USA

## Abstract

The 2022 Supreme Court ruling in *Dobbs v. Jackson Women′s Health Organization* returned regulatory authority over abortion to the states and precipitated a wave of restrictive state‐level legislation. This narrative review examines how these state responses have influenced medical residency applications, physician training, and maternal health equity. Existing research indicates that restrictive state laws have contributed to a decline in residency applications to programs in jurisdictions with near‐total abortion bans. A growing body of evidence suggests that many medical students, particularly those seeking OBGYN training, prioritize programs in states with fewer restrictions to ensure comprehensive clinical education. These trends may exacerbate existing maternity care deserts by contributing to physician shortages, particularly in underserved and rural areas. The review further explores how the post‐*Dobbs* regulatory environment impacts physician training. Many OBGYN residency programs in restrictive states struggle to provide abortion training, often requiring residents to travel out of state, which creates significant financial and logistical burdens. Additionally, a lack of transparency regarding training availability in these states complicates the ability of prospective residents to assess program offerings. Beyond residency applications, these state‐level restrictions have affected practicing physicians, contributing to burnout, legal uncertainties, and ethical dilemmas. Many providers in restrictive jurisdictions report moral distress and legal risks when making clinical decisions, leading some to relocate to states with greater reproductive freedoms. The compounded effects of these laws may widen healthcare disparities, disproportionately affecting marginalized communities, particularly low‐income individuals and people of color. Given the limited longitudinal evidence since *Dobbs*, continued monitoring of OBGYN residency applications, training availability, workforce distribution, and maternal health outcomes is needed to determine the persistence and magnitude of these effects.

## 1. Introduction

From the formation of the American Medical Association (AMA) in 1847 to the landmark 1973 ruling in *Roe v. Wade* and its subsequent overturning in 2022, abortion has remained a centerpiece of legal and ethical controversy in the United States [1]. The initial push for legalization was driven largely by rising mortality rates from unsafe, clandestine procedures; the Guttmacher Institute found that in 1930 alone, illegal abortions accounted for one in five maternal deaths [2]. Even before federal legalization in 1973, Hawaii, California, New York, and Alaska had repealed their abortion bans, illustrating that reproductive healthcare policies and attitudes have historically been heterogeneous across state lines.

Physicians and the broader medical community have consistently been impacted by and have actively shaped these legislative shifts. Although the AMA was involved in the initial criminalization of abortion in the mid‐19th century, the profession later became instrumental in reform efforts. A pivotal moment was the “Trial of the San Francisco Nine” in 1966, in which nine prominent obstetricians faced the revocation of their medical licenses by the California Board of Medical Examiners. These physicians had performed hospital‐approved abortions for patients exposed to a rubella epidemic, citing the high risk of severe fetal anomalies, a practice that, while medically accepted, was technically illegal under California′s 1872 antiabortion statute. The case became a “catalyst for change” because it shifted the debate from criminal activity to professional medical judgment. The “Nine” received unprecedented support from the academic medical community; the deans of 128 out of 134 US medical schools signed an *amicus curiae* brief on their behalf, arguing that medical education and practice must be governed by clinical evidence rather than rigid, antiquated statutes [1]. This defense successfully framed abortion as a component of comprehensive medical care, ultimately leading to California′s Therapeutic Abortion Act of 1967 and cementing the historical link between abortion access, physician autonomy, and the integrity of medical education.

Following the Supreme Court′s decision in *Planned Parenthood v. Casey* (1992), state‐level abortion laws became increasingly restrictive through the introduction of Targeted Regulation of Abortion Providers (TRAP) laws. These statutes imposed burdensome and often medically unnecessary administrative and structural requirements on facilities, such as mandating that clinics meet the same physical standards as ambulatory surgical centers or requiring physicians to hold local hospital admitting privileges, which effectively forced many clinics to close.

More recently, Texas served as an early case study for the medical and legal impacts of abortion prohibition through the implementation of Senate Bill 8 (SB 8) in September 2021. SB 8 banned abortion after the detection of embryonic cardiac activity at approximately 6 weeks of gestation and employed a novel private civil enforcement mechanism that allowed any individual to sue those who “aid or abet” an abortion. Because SB 8 effectively ended most abortion access in the state nearly 10 months before the 2022 Supreme Court ruling in *Dobbs v. Jackson Women′s Health Organization*, it provided a preliminary window into the “chilling effect” on medical practice and residency training. Crucially, the *Dobbs* decision did not itself ban abortion; rather, it eliminated the federal constitutional protection for the procedure and returned full regulatory authority to the states. This jurisdictional shift occurred alongside a growing population of people who can become pregnant and an intensifying shortage of OBGYN providers [3].

The mediated effects of these subsequent state‐level restrictions have created significant hurdles for medical education. Although the Accreditation Council for Graduate Medical Education (ACGME) requires all OBGYN residency programs to provide access to abortion training, nearly half of all residents now train in states with restrictive laws [4]. In these jurisdictions, residents must often travel out of state to meet clinical requirements, incurring substantial financial and logistical burdens. Furthermore, a survey of third‐ and fourth‐year medical students conducted shortly after the *Dobbs* ruling found that 76.9% of respondents reported that changes in abortion access would influence their choice of residency specialty and geographic location [5].

Current data supports these concerns; a 2024 cross‐sectional study revealed a significant decrease in residency applications to programs in states with the strictest abortion laws [4]. Given that the majority of designated “maternity care deserts” are already located in restrictive states, the ongoing decline in applicants may further shrink the provider workforce and worsen maternal health outcomes [6]. The purpose of this literature review is to examine how the post‐*Dobbs* regulatory landscape has influenced residency training preferences and how these shifts contribute to burgeoning healthcare disparities for patients residing in underserved areas.

## 2. Methods

This narrative review is aimed at synthesizing current evidence on how abortion policy changes affect medical education and the OBGYN workforce. The authors conducted a systematic search of the PubMed/MEDLINE, Scopus, and Google Scholar databases to identify relevant peer‐reviewed studies. The search strategy utilized combinations of MeSH terms and keywords, including: “Dobbs v. Jackson,” “medical residency applications,” “OBGYN training,” “abortion restrictions,” and “maternal health equity.”

The search was conducted throughout February 2025 and concluded on March 1, 2025. To ensure relevance to the current sociopolitical and legal climate, studies were eligible for inclusion if they were published in or after 2020. Exclusion criteria included studies not available in English, those focusing solely on international healthcare systems without a US comparison, and commentaries or editorials that did not present original data or systematic syntheses. Grey literature, including policy briefs from the Guttmacher Institute and the Association of American Medical Colleges (AAMC) reports, was systematically included to capture emerging data in this rapidly evolving landscape.

The screening process was managed using Covidence, a web‐based tool for systematic reviews. The first three authors independently screened titles and abstracts, followed by a full‐text review to ensure organization and minimize selection bias. Each reviewer worked through the references separately and remained blinded to the votes of the other two reviewers during the initial screening phase; discrepancies were resolved through consensus. Following this process, 14 studies remained eligible for analysis. Data and key findings, including study design, geographic focus, and primary outcomes, were systematically extracted and synthesized for this review.

## 3. Results

### 3.1. Influence of Abortion Restrictions on Residency Program Selection and Practice Locations

*Dobbs v. Jackson Women′s Health Organization* has impacted future and current physicians in all specialties in several ways. Laws regarding abortion access impact where medical students and residents choose to complete their training to become physicians, the education and training they receive, and how and where they choose to practice once they are trained physicians. Medical students have some freedom to choose where they complete their residency training to become physicians, along with which specialty they want to train in. Between 6 August and 22 October 2022, third‐year and fourth‐year US medical students applying to US residency programs were surveyed by Traub et al. to examine how *Dobbs* is influencing the ideological, personal, and professional factors they must reconcile when choosing where and how to complete residency. Medical students entering residency, especially those who can become pregnant, recognize that their existing lack of autonomy may be exacerbated by changes in access to reproductive healthcare. Respondents in this study highlighted *Dobbs* as a major factor impacting their decision on where to apply for residency, indicating the significant influence abortion access is having on where the next generation of physicians will be training. The respondents applying only to programs in states with abortion restrictions predominantly identified as Catholic and cisgender male [7]. Although this survey reflects the opinions of medical students applying to any specialty, the trend in those students applying to OBGYN residency programs demonstrated a clear shift in geographic distribution in the year following abortion access changes. Hammoud et al. conducted a comprehensive review examining the impact of the *Dobbs v. Jackson Women′s Health Organization* on healthcare providers and medical training and found that although overall applicant numbers remained stable between 2019 and 2023, the distribution of applicants differed significantly by state abortion ban status in the 2022 and 2023 match cycles when comparing data from 2019 through 2023. There was a 15% decrease in OBGYN residency applications in restrictive states post‐*Dobbs*, as 44% of residency programs in restrictive states now have limited access to abortion training. Eighty‐two percent of students preferred states with fewer restrictions for training, which may contribute to shifts in workforce distribution in the future [4].

Woodcock et al. also examined how restrictions may affect OBGYN residents′ plans after graduation and their transition to practice. Their mixed‐methods study surveyed graduates from programs affiliated with the Kenneth J. Ryan Residency Training Program in Abortion and Family Planning, a national initiative founded in 1999 to help residency programs meet ACGME requirements for opt‐out family planning and abortion training. By focusing on these 109 Ryan Program sites between March and April 2023, the researchers captured the perspectives of trainees specifically prepared for comprehensive reproductive care. Regardless of their training site, 17.6% of these residents changed their intended practice location after the *Dobbs* decision, a shift directly linked to state‐level abortion restrictions and personal access to care. Residents who had originally intended to practice in restrictive states were 8.52 times more likely to change their future location post‐*Dobbs*, with 82.0% expressing strong advocacy for abortion access. Furthermore, only 33.4% maintained plans to work in restrictive jurisdictions. This trend extended into subspecialty training; many residents ranked fellowship programs in restricted states lower or avoided applying altogether, particularly in fields like reproductive endocrinology [8]. Thus, the post‐*Dobbs* regulatory environment has not only influenced initial residency selection but has created a provisional trend of workforce redistribution as newly trained physicians, specifically those from Ryan Program‐affiliated institutions, opt to establish their practices in states with fewer legal constraints.

*Dobbs* also influences the type of training that medical students and residents receive during their education. Before the 2022 ruling, Vinekar et al. predicted that 44.8% of accredited OBGYN residency programs were in states that were certain or likely to ban abortion if *Roe v. Wade* was overturned, thus impacting residency training. They also predicted 43.9% of the 2022 OBGYN residents were projected to lack access to in‐state abortion training, highlighting significant potential challenges for future training and workforce development in the field [9]. Following the overturning of *Roe v. Wade*, these projections largely materialized. Beasley et al. found that in 2024, 17% of medical schools do not offer formal abortion education, and only 44% provide clinical training, reducing the number of trained providers. Only 64% of OBGYN residency programs offer dedicated abortion training, with many facing state and institutional barriers. Even with specialized fellowship programs, physicians still face significant barriers to providing abortion care, especially in restrictive states. Nearly 50% of OBGYN residency programs and 40% of residents are now in states that have banned or restricted abortion access, which complicates training for abortion care. Programs in restricted states are coordinating with programs in protected states for abortion training, but logistical and legal challenges are increasing, requiring careful coordination for out‐of‐state rotations [10]. Additionally, for medical students applying to OBGYN residency programs, it is difficult to even find this information about abortion training. In a 2024 study, Karlapudi et al. aimed to characterize public info on abortion training in OBGYN residency programs in states with and without bans. After a systematic internet search of every program′s website to identify info on abortion training, it was found that two‐thirds of OBGYN residency programs provide public information about abortion training, but half of the programs in abortion‐restricted states do not. Residency programs in states with legal abortion are more likely to offer publicly available information and Ryan Program affiliation. Many programs avoid using the term “abortion” and instead refer to “family planning” when describing training, making it unclear if abortion training is included. Applicants may struggle to identify programs offering abortion training, especially in restrictive states, underscoring the need for greater transparency [11]. The training received by OBGYN residents in all states has been significantly impacted by the *Dobbs* ruling and subsequent state‐level legal responses. These current disruptions to training may have longer‐term effects on physician education and workforce distribution if they persist over time.

Finally, for both newly trained and practicing attending physicians, the post‐*Dobbs* regulatory environment has significantly impacted how they practice medicine and, subsequently, how patient care has been affected, and this goes well beyond just the field of OBGYN. Roper et al. added 10 questions to the 2022 Council of Academic Family Medicine Educational Research Alliance general membership survey to evaluate the impact on relevant themes in reproductive health care and training after the *Dobbs* decision. Responses were categorized by the severity of restriction of state abortion policies. The 991 respondents with clinical responsibilities (82% of the total respondents) reported significant changes in their counseling practices, clinical decision making, worry of legal risks, and trust in patients′ self‐reported reproductive medical history, compared with peers in protective states. Respondents in restrictive states also reported decreased confidence in family medicine residency programs and perceived them as less desirable [12]. Brindis et al. examined how *Dobbs* impacted the entire national medical workforce. Impacts vary across states, but providers in states with restrictive abortion laws now must contend with evolving legal and ethical challenges that have the potential to affect physicians through workforce safety, mental health, education, and training opportunities. Additionally, some state abortion laws may conflict with federal statutes such as the Emergency Medical Treatment and Active Labor Act (EMTALA), exposing providers to legal uncertainty and potential penalties. The *Dobbs* decision may increase burnout and moral distress among providers, particularly those in restrictive states, further affecting residency applications, training opportunities, and long‐term workforce trends [13]. These effects extend beyond medical trainees and decisions on where to practice, but also to the currently established physicians in all states.

### 3.2. Persistent Gaps in Reproductive Healthcare and Their Impact on Access, Equity, and Maternal Health Outcomes

The American College of Obstetricians and Gynecologists (ACOG) has consistently emphasized that legislative restrictions on abortion services interfere with the patient–health care professional relationship and disproportionately affect individuals with low incomes and those residing in underserved areas [14]. This aligns with the Zhu et al. scoping review of 54 studies, which found that post‐*Dobbs* abortion bans have led to clinic closures and forced patients to travel over 400 mi for care. These increased travel distances serve as a primary mechanical barrier to equity, as the associated financial and logistical burdens fall most heavily on marginalized populations. Additionally, the study noted a 21% increase in demand for permanent contraception in restrictive states and heightened concerns among medical trainees regarding legal risks, contributing to potential provider shortages. The review underscores how these restrictions exacerbate inequities in maternal healthcare access and outcomes, particularly in areas already facing provider shortages and limited healthcare infrastructure [15].

Further supporting these concerns, a 2024 study by Morin et al. investigates the impact of restrictive abortion laws on the OBGYN workforce in Texas, revealing that the attrition of providers directly correlates with longer wait times for prenatal services and reduced access to emergency obstetric care. This study highlights that Black and Indigenous patients, who already navigate a healthcare system with a baseline of higher maternal mortality, are uniquely vulnerable to these workforce shifts. Specifically, the relocation of OBGYNs from restrictive jurisdictions removes the last line of defense for patients experiencing high‐risk complications such as preeclampsia or postpartum hemorrhage. For these populations, the loss of a local provider does not just mean a longer commute; it often results in the total absence of life‐saving intervention during crucial hours of obstetric emergencies [16].

Kheyfets et al. examine how restrictive abortion laws contribute to the worsening maternal mortality crisis in the United States, particularly in states where the legal environment restricts clinical education. Their analysis reveals that states with more restrictive abortion policies tend to have significantly higher maternal mortality rates, with Black pregnant individuals facing the greatest disparities due to systemic barriers and reduced access to care. States with the most restrictive policies report significantly higher maternal mortality rates, with Black pregnant individuals facing the greatest disparities. This is not merely a geographic coincidence but a mediated outcome of systemic barriers. As providers leave or limit their practice due to legal uncertainty, the density of skilled clinicians capable of managing complex pregnancies thins, leaving marginalized patients to rely on overextended emergency departments that may lack specialized obstetric expertise. Additionally, the authors highlight the role of misinformation and lack of abortion education in worsening health outcomes, emphasizing the urgent need for policy reforms to address these inequities and improve maternal health [17].

Wallace et al. further elucidate the challenges faced in regions with limited access to maternity care, even before the overturning of *Roe v. Wade*. Analyzing data from Louisiana, the researchers found that certain Louisiana parishes lacking adequate maternal health services experienced significantly higher rates of pregnancy‐associated mortality. This correlation underscores the compounded risks in regions where restrictive abortion laws coincide with insufficient healthcare infrastructure, leading to poorer maternal health outcomes [6]. These restrictions also exacerbate provider shortages, a concern further highlighted by Mermin‐Bunnell et al., who found that medical students are increasingly deterred from applying to residency programs in states with abortion bans. This shift threatens to deepen existing gaps in reproductive healthcare by reducing the number of trained providers in states already facing critical shortages. By discouraging new physicians from entering the pipeline in restrictive jurisdictions, these policies have the potential to create a structural deficit in the workforce, and the geographic and racial gaps in maternal health outcomes may continue to widen as the next generation of providers clusters in states with greater reproductive freedoms. As fewer physicians choose to practice in restrictive states, patients encounter longer wait times, fewer available specialists, and increased financial and logistical burdens when seeking care [5].

## 4. Discussion

The jurisdictional shift following the *Dobbs* (2022) decision and the subsequent enactment of restrictive abortion laws in several US states have reshaped the landscape of medical residency applications, physician training, and healthcare access. This literature review synthesizes a growing body of evidence showing that state‐level legislative responses, mediated by the removal of federal constitutional protections, are influencing residency selection and training. These findings are largely consistent with the existing literature on the intersection of healthcare access and law, but the full extent of these implications remains to be seen.

The studies reviewed reveal several clear trends: firstly, restrictive state laws, rather than the *Dobbs* ruling in isolation, have influenced medical students′ decisions regarding where to train. Following the implementation of state‐level bans enabled by *Dobbs*, there has been a notable decline in residency applications to programs in those jurisdictions. This aligns with research indicating students increasingly consider local legal environments when choosing training sites. Studies by Hammoud et al. and Woodcock et al. further suggest residents in restrictive regulatory environments may be more likely to change their practice location after graduation. These shifts appear linked to statutory constraints governing clinical practice rather than to the Court decision alone.

A strength of this review lies in its mapping of diverse study designs. However, a primary limitation is the inability to compare long‐term data across eras. The period since *Dobbs* returned regulatory authority to the states is only 2.5 years old, compared with nearly 50 years of research under the *Roe* and *Casey* frameworks. Consequently, the long‐term impacts of this newly fragmented legal landscape on workforce distribution and maternal health remain a subject of active, ongoing study.

The implications for health equity are far‐reaching and extend beyond the physician workforce. The thinning of the OBGYN pipeline creates a secondary “ripple effect” that disrupts the entire multidisciplinary care team, including genetic counselors, social workers, and labor‐and‐delivery nursing staff. In restrictive jurisdictions, these providers often face similar ethical dilemmas and legal uncertainties when navigating complex fetal diagnoses or social support for patients requiring out‐of‐state travel. This systemic erosion of the specialized workforce, where the loss of an OBGYN often leads to the underutilization or relocation of affiliated support staff, further exacerbates disparities in maternal health outcomes. For Black and Indigenous patients, the loss of these culturally competent navigators is particularly devastating, as these providers are often the primary link between the patient and the increasingly fragmented healthcare infrastructure. Additionally, state‐specific restrictions may contribute to provider shortages, especially in rural areas. This pattern underscores how the varied legislative landscape post‐*Dobbs* may further entrench geographic disparities in health outcomes.

## 5. Conclusion

The *Dobbs v. Jackson* (2022) ruling, which returned abortion jurisdiction to state legislatures, has profound implications for the future of the medical workforce. This review underscores the impact of post‐*Dobbs* state laws on OBGYN residency applications and physician retention. As discussed, recent data demonstrate a decline in applications in states with near‐total bans; for example, Texas reported a 16% decline in applications following the implementation of state‐level restrictions. Additionally, provider shortages are exacerbated by physicians opting to leave these restrictive jurisdictions or forgoing obstetrics to avoid legal uncertainty. Although the *Dobbs* decision served as the legal catalyst, the primary drivers of these shifts are the heterogeneous state‐level regulations that followed. Given the limited 2.5‐year data set, continued longitudinal research is necessary to track whether these mediated workforce trends will lead to lasting changes in the geographic distribution of OBGYN providers.

## Author Contributions

Brooke Gully and Ellie Whitney are contributing first authors.

## Funding

No funding was received for this manuscript.

## Conflicts of Interest

The authors declare no conflicts of interest.

## Data Availability

Data sharing is not applicable to this article as no datasets were generated or analyzed during the current study.
